# Long-term outcomes of transvaginal mesh surgery for pelvic organ prolapse: a retrospective cohort study

**DOI:** 10.1186/s12905-021-01505-z

**Published:** 2021-10-11

**Authors:** Xiaojuan Wang, Yisong Chen, Changdong Hu, Keqin Hua

**Affiliations:** Department of Gynecology, The Obstetrics and Gynecology Hospital of Fudan University, 128 Shenyang Road, Shanghai, China

**Keywords:** Pelvic organ prolapse, Transvaginal mesh, Long-term follow-up

## Abstract

**Background:**

The objective of this study was to evaluate the overall outcomes and complications of transvaginal mesh (TVM) placement for the management of pelvic organ prolapse (POP) with different meshes with a greater than 10-years of follow-up.

**Methods:**

We performed a retrospective review of patients with POP who underwent prolapse repair surgery with placement of transvaginal mesh (Prolift kit or self-cut Gynemesh) between January 2005 and December 2010. Baseline of patient characteristics were collected from the patients’ medical records. During follow-up, the anatomical outcomes were evaluated using the POP Quantification system, and the Patient Global Impression of Improvement (PGI-I) was used to assess the response of a condition to therapy. Overall postoperative satisfaction was assessed by the following question: “What is your overall postoperative satisfaction, on a scale from 0 to 10?”. Relapse-free survival was analyzed using Kaplan–Meier curves.

**Results:**

In total, 134 patients were included. With a median 12-year (range 10–15) follow-up, 52 patients (38.8%) underwent TVM surgery with Prolift, and Gynemesh was used 82 (61.2%). 91% patients felt that POP symptom improved based on the PGI-I scores, and most satisfied after operation. The recurrence rates of anterior, apical and posterior compartment prolapse were 5.2%, 5.2%, and 2.2%, respectively. No significant differences in POP recurrence, mesh-associated complications and urinary incontinence were noted between TVM surgery with Prolift versus Gynemesh.

**Conclusions:**

Treatment of POP by TVM surgery exhibited long-term effectiveness with acceptable morbidity. The outcomes of the mesh kit were the same as those for self-cutmesh.

## Background

Since gynecologists began using mesh for surgical treatment of stress urinary incontinence (SUI) as well as transvaginal repair of pelvic organ prolapse (POP) in the mid-1990s [1], some randomized controlled trials have reported the effectiveness of mesh surgery compared with traditional repairs [2, 3]. However, the reports of mesh-related complications are increasing [4]. Over the last decade, the Food and Drug Administration has issued warnings, reclassified transvaginal mesh (TVM) from a class II to a class III device, and recently ordered the cessation of sales and distribution of transvaginal mesh [5]. Despite a decrease in the use of TVM used for POP repair surgery, the risk of mesh-associated complications has not diminish.

Previous publications reported that the long-term outcomes of surgical treatment of POP with mesh offered low recurrence rates, better satisfaction, and high cumulative reoperation and mesh exposure rates [6–8]. Most publications reported the outcomes based on 4–5 years of follow-up [7, 9, 10], and only few publications with small sample sizes reported the outcomes at greater than 10 years after mesh repair surgery [6]. Here, we aimed to evaluate the outcomes following synthetic mesh placement by the vaginal route for POP with greater than 10 years of follow-up in a larger group, and to compare the outcomes of pelvic floor repair with different meshes.

## Methods

In this single-center retrospective study, the medical records of women who underwent surgical transvaginal treatment for POP using Gynemesh (Ethicon, Somerville, NJ) or Prolift kit (Gynecare, Somerville, NJ) between January 2005 and December 2010 were reviewed. Our institution is a tertiary university-affiliated hospital that perform a high volume of surgeries. Baseline clinical characteristics, and perioperative data such as concomitant procedures, surgical complications, and readmission, were recorded from the electronic medical record system of our hospital. The severity of POP was defined using the Pelvic Organ Prolapse Quantification (POP-Q) system [11]. The following inclusion criterion was employed: patients were suffering from at least symptomatic stage 2 to 4 POP of any compartment. The exclusion criteria were incomplete pre or postoperative data and mental illness.

The Prolift system was a precut, commercial kit, and Gynemesh was a single 15 × 10 cm piece, that was cut into two parts (four arms and joint of the anterior mesh, another of rectangular strips) [12]. Both were porous, monofilament woven polypropylene mesh. The price was the greatest difference between the two types of mesh. The later was less expensive than the former. If the patients underwent repair using Gynemesh, the surgery was done as previous described [13]. If the patients underwent repair using the Prolift procedure, the surgery was performed as described by Fatton et al. [14].

### Surgery procedure

A midline vertical full-thickness anterior vaginal incision was made from 1 to 1.5 cm below the urethral meatus and extended toward the apex. The bladder was dissected from the vagina toward the inferior pubic ramus until the arcus tendineus fascia pelvis (ATFP) was reached bilaterally. The commercial mesh (kit or self-cut) was placed with the use of four needle passages. Two skin incisions were made on both sides: 1 cm lateral to the urethral meatus and ramus of pubis descending and 2 cm below and 1 cm lateral to the first incision for the passage of the needles. The needles were inserted using the transobturator approach and the obturator membrane was perforated at the level of the ATFP. The vaginal epithelium was not trimmed and was closed with a nonlocking continuous suture. The posterior vaginal wall epithelium was opened in the midline and dissected laterally until the sacrospinous ligament could be palpated. Two skin incisions were made on both sides: 3 cm lateral and 3 cm inferior to the anus. The needles were punctured through the anorectal fossa, and through the sacrospinous fascia and the spine fascia near the ischial spine. The mesh remained in place without tension, and the vaginal mucosa was closed without trimming.

A concomitant vaginal hysterectomy was performed in some patients, and concomitant anti-continence surgery was administered to the patients who were diagnosed with stress urinary incontinence and required anti-continence management.

The follow-up was performed in a standardized manner as part of regular practice: urogynecological physical examination and POP-Q were performed by two experienced urogynecologist at 1, 6, 12 months, followed by annually thereafter. The patients who did not come to outpatient follow-up visit were contacted by phone. Recurrence was defined as ≥ stage 2 POP. Mesh-associated complications included mesh vaginal extrusion, vaginal bleeding, and pain (pelvic pain or dyspareunia). Urinary tract infection was also recorded. The last date of follow-up visit was defined as the date of last follow-up. The Patient Global Impression of Improvement (PGI-I) [15] was used to assess the response of a condition to therapy during the last follow-up. Overall postoperative satisfaction was assessed using a 10-point visual analog scale [16], with 0 = poor, and 10 = excellent, by asking “What is your overall postoperative satisfaction, on a scale from 0 to 10?”.

### Statistical analyses

We calculated either the means and standard deviations or the medians and ranges for continuous variables as well as the frequencies (percentages) for categorical variables. Continuous variables were compared with the t-test or Wilcoxon test according to the distribution, and categorical variables were compared with the chi-squared test or Fisher's test, according to the assumptions. The cumulative proportion of relapse-free patients during follow-up was analyzed by Kaplan–Meier curves, and group comparisons were analyzed by log-rank. A *p* < 0.05 was considered statistically significant. SPSS software (SPSS version 22.0, 2013; SPSS Inc, Chicago, IL) was used to perform statistical analyses.

## Results

**Fig. 1:**
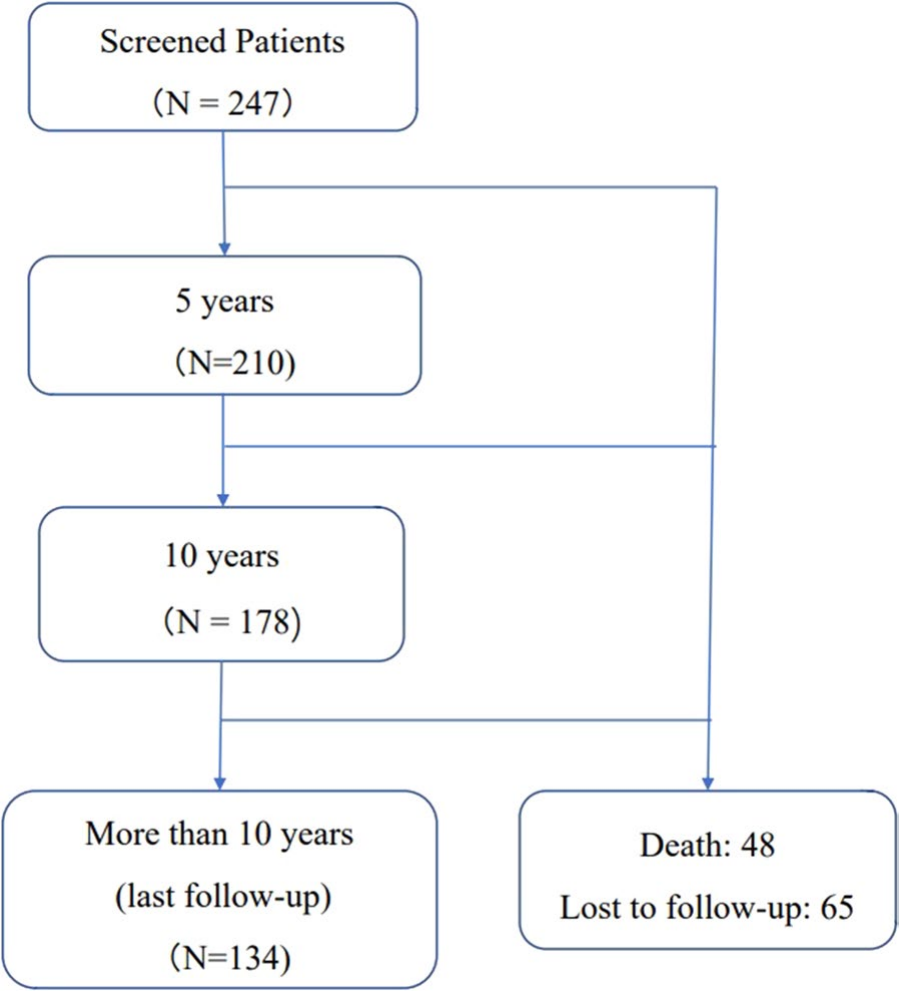
Flow chart of follow-up

In total, 247 patients were initially included in this study. During follow-up (Fig. 1), the cumulative death rate was 19.4% (48 out of 247), and no death was related to surgery complications. The overall loss to follow-up rate was 26.3% (65 out of 247). In total, 210 patients were included in the 5 year follow-up analysis, and 134 patients were included in the last follow-up.

During the last follow-up, 52 patients (38.8%) were given TVM with Prolift kit, and 82 patients (61.2%) were given self-cut Gynemesh. Most of the patients underwent total repair. In total, 3 of 52 patients (5.8%) underwent anterior repair with the Prolift kit, and 8 of 82 patients (15.4%) underwent anterior repair with Gynemesh. No patients underwent posterior repair. The mean number of years follow-up was 11.8 (± 1.32), with a median of 12 years (range 10–15). The median age was 75 years (range 42–93). The preoperative patient characteristics are shown in Table 1. The preoperative median age was 62.1 years (range 29–80), and no significant differences were noted between the Prolift kit and Gynemesh groups. Greater than half of the patients experienced advanced anterior/apical vaginal wall prolapse, and 23.9% of the patients were diagnosed with advanced posterior vaginal wall prolapse. 18 patients (13.4%) experienced urinary incontinence complications (including stress urinary incontinence, urge incontinence, mixed incontinence), and 6 patients (4.5%) underwent anti-incontinence surgery with a sling. The uterus was preserved in19 patients (14.2%). No differences were noted between Prolift group and Gynemesh group. Two patients with Prolift experienced bladder injury during operation, and one patient with Gynemesh experienced bladder injury during operation. Three patients with Gynemesh experienced readmission because of vaginal bleeding, one patient with Prolift experienced readmission because of urinary intention, and one patient with Prolift readmitted because of vaginal bleeding.

**Table 1:** Preoperative patients’ characteristics

Preoperative patients’ characteristics (n = 134)					
Mesh		Total (n = 134)	Prolift (n = 52, 38.8%)	Gynemesh (n = 82, 61.2%)	*P*-value
Age (years), median		62.1 (29–80)	63.2 (45–79)	61.4(29–80)	0.131
BMI (kg/m^2^), median		24.59 (17.78–30.04)	24.92 (17.78–29.40)	24.38 (19.92–30.04)	0.800
Anterior compartment prolapse stage (POP-Q), n (%)	3–4	99 (73.9%)	39 (75%)	60 (73.2%)	0.814
Apical compartment prolapse stage (POP-Q), n (%)	3–4	91 (67.9%)	39 (75%)	52 (63.4%)	0.162
Posterior compartment prolapse stage (POP-Q), n (%)	3–4	32 (23.9%)	17 (32.7%)	15 (18.3%)	0.057
Parity, median		2.16 (1–6)	2.12 (1–6)	2.23 (1–5)	0.430
Urinary Incontinence	Any kind, n (%)	18 (13.4%)	5 (9.6%)	13 (15.9%)	0.302
Hysterectomy history, n (%)		3 (2.2%)	2 (3.8%)	1 (1.2%)	0.317
Concomitant MUS, n (%)		6 (4.5%)	3 (5.8%)	3 (3.7%)	0.565
Preserved Uterine, n (%)		19 (14.2%)	6 (11.5%)	13 (15.9%)	0.485

Continuous variables were compared with—test, categorical variables with Chi-Square test

*POP-Q*, pelvic organ prolapse quantification system; *MUI*, mixed urinary incontinence; *MUS*, mid-urethral sling

Regarding the feeling of POP symptom improvement/worsening, the following PGI-I scores were recorded at the last follow-up: PGI-I 1 to 3 (improvement), 122 out of 134 (91%); PGI-I 4 (no change), 3 out of 134 (2.2%); PGI-I 5–7 (worsening): 9 out of 134 (6.7%). The median answer to the question “What is your overall postoperative satisfaction, on a scale from 0 to 10?” was 8 (range 6 to10).

**Fig. 2:**
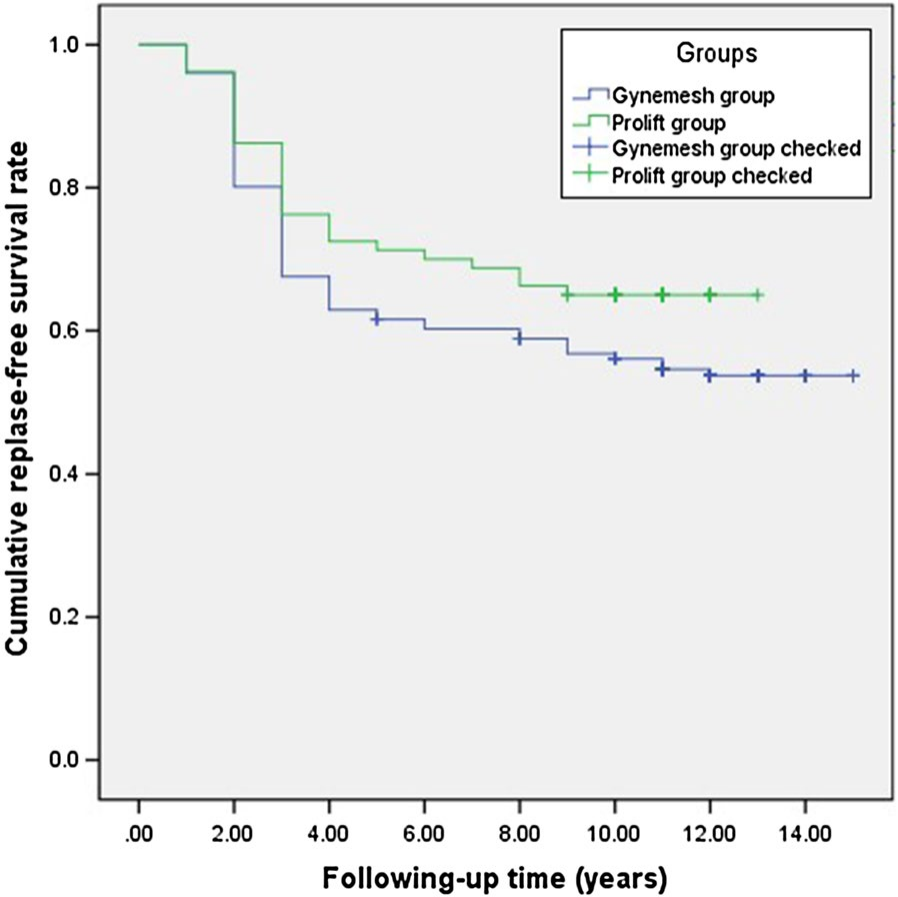
Relapse-free survival rate between Prolift and Gynemesh groups

We compared the outcomes of different mesh group based on the period of follow-up, and found that 94 patients were given TVM with a Prolift kit, and 116 patients were given self-cut Gynemesh during the 5-year follow-up. No significant differences in recurrence, mesh-associated complications or urinary incontinence were noted between both the groups. During the last follow-up, 52 patients were given TVM with Prolift, and 82 patients were given with Gynemesh. Similarly, no significant differences in recurrence, mesh-associated complications or urinary incontinence were noted between the groups (Table 2). No differences (*p* = 0.142) in the cumulative relapse-free survival rate was noted between the two groups (Fig. 2).

**Table 2:** Follow up patients’ characteristics stratified by type of mesh

Follow up patients’ characteristics		5 Years follow up characteristics (n = 210)		Comparison	Last follow up characteristics (n = 134)		Comparison
Mesh		Prolift (n = 94)	Gynemesh (n = 116)	*P* value	Prolift (n = 52)	Gynemesh (n = 82)	*P* value
Anterior Vaginal Wall prolapse stage (POP-Q), n (%)	0–1	87 (92.5%)	110 (94.8%)	0.496	49 (94.2%)	78 (95.1%)	0.821
	≥ 2	7 (7.4%)	6 (5.2%)		3 (5.8%)	4 (4.9%)	
Apical Vaginal Wall prolapse stage (POP-Q), n (%)	0–1	89 (94.7%)	104 (89.7%)	0.184	50 (96.2%)	78 (95.1%)	0.778
	≥ 2	5 (5.3%)	12 (10.3%)		2 (3.8%)	5 (4.9%)	
Posterior Vaginal Wall prolapse stage (POP-Q), n (%)	0–1	91 (96.8%)	111 (95.7%)	0.674	51 (98.1%)	80 (97.6%)	0.844
	≥ 2	3 (3.2%)	5 (4.3%)		1 (1.9%)	2 (2.4%)	
Repeated POP Surgery, n (%)		1 (1.1%)	3 (2.6%)	0.422	2 (3.8%)	3 (3.7%)	0.955
Mesh vaginal extrusion, n (%)		5 (5.3%)	6 (5.2%)	0.962	5 (9.6%)	7 (8.5%)	0.831
Vaginal bleeding, n (%)		2 (2.1%)	2 (1.7%)	0.832	3 (5.8%)	4 (4.9%)	0.821
Pain#, n (%)		4 (4.3%)	7 (6.0%)	0.565	2 (3.8%)	3 (3.7%)	0.955
Urinary Incontinence, n (%)		13 (13.8%)	11 (9.5%)	0.325	3 (5.8%)	5 (6.1%)	0.938
Urinary tract infection, n (%)		5 (5.3%)	7 (6.0%)	0.824	3 (5.8%)	6 (7.3%)	0.727
Subsequent MUS surgery, n (%)		5 (5.3%)	3 (2.6%)	0.304	1 (1.9%)	3 (3.7%)	0.565

^#^Pelvic pain or dyspareunia

## Discussion

Due to the lower risk of recurrence, TVM for POP had been widely used for POP more than a decade ago. However, the higher rates of surgical complications and postoperative adverse events [17] resulted in the withdraw of vaginal mesh kits from the market. In 2018, the United Kingdom government ordered a temporary restriction on the use of vaginal meshes, and Ireland has also taken a similar decision; in 2019, FDA ordered all manufacturers of meshes for vaginal surgery to immediately stop selling and distributing their products in the United States due to insufficient evidence of safety [18, 19].

However, some retrospective studies showed that last-generation mesh (Uphold™ mesh) for POP with long follow-up resulted in low complication and reoperation rates [20, 21], which argued against abandoning vaginal mesh use for POP. With an average of greater than 10 years of follow-up, our study showed very good functional outcomes (PGI-I 1 to 3 [improvement]: 91%), and the median answer to the question “What is your overall postoperative satisfaction, on a scale from 0 to 10?” was 8 (range 6–10), which was consistent with most publications [6, 22].

Our study found low recurrence rates after pelvic floor repair with mesh (Prolift kit or self-cut Gynemesh). The current publications showed low recurrence after greater than 5 years and less than 10 years of follow-up [9, 23, 24]. We found that the low recurrence rates of anterior, apical and posterior compartment prolapse were 5.2%, 5.2%, 2.2% respectively, after greater than 10 years of follow-up. Given the limited number of publications on the outcomes with considerably long-term follow-up, this phenomenon might be due to the greater lost to follow-up, or the formation of integration, leading to continuous mechanical support. With longer-term follow-up and more similar publications, we should gain more information on outcomes of vaginal mesh repair for POP.

However, the mesh-associated complication rate during the last follow-up was greater than that at the 5-year follow-up. Many publications have compared the complications of mesh repair with those of native tissue repair, demonstrating significantly higher complication rates of mesh repair compared with those of nonmesh procedures [8–10, 25]. Few publications have compared the mesh-related complications based on the follow-up period. One publication with long-term follow-up reported 25% mesh exposure (16 out of 63) [6]; however, this study did not describe when complications were occurred. What is the explanation for the increased complication rate over time? Age was a high risk factor for POP [26], and the vaginal mucosa becomes thinner with age. Studies on the effect of age on vaginal wound healing showed that excessive and prolonged macrophage response in older rats may contribute to poor wound healing in the vagina [27, 28]. Therefore, mesh-related complications should not decrease in response to the removal of transvaginal mesh from the market, and more complications will likely be encountered in the future. Persistent vaginal bleeding, vaginal discharge, or recurrent urinary tract infections after mesh placement might be due to mesh erosion, and further evaluation of exposure or erosion should be performed [29]. Asymptomatic exposure of monofilament microporous meshes can be managed expectantly with vaginal estrogen.

Although the Gynemesh was cut into different parts for use in pelvic floor reconstruction, our study also revealed no significant difference in outcomes between the Prolift kit group and the self-cut Gynemesh. It has been reported that mesh kits are not related to perioperative surgical complication rate, or to subjective or objective outcomes, therefore suggesting that the type and shape of polypropylene mesh is not associated with outcomes or complications [7]. Our study revealed a low complications rate, low POP recurrence rate and high subjective satisfaction during the a very long-term follow-up. These findings were consistent with the finding that surgical expertise was a more important predictive factor than the mesh itself for postoperative functional and anatomical outcomes [7, 30, 31].

The following strengths of this study are noted: first, our study is one of the few studies to report the outcomes of TVM surgery for POP with an extremely long-term follow-up; second, all surgeries were performed in a standardized manner by experienced surgeons, which eliminated variability in surgical technique as a confounder. However, our study was limited due to its retrospective nature, which was subject to measurement and selection bias. Another limitation was the high rate of loss to follow-up. Unfortunately, the longer the follow-up, the greater the rate of loss to follow-up [6, 32].

## Conclusion

At very long-term follow up, the recurrence rate after pelvic repair surgery with mesh for POP remained low and the subjective satisfaction rate was high. Although, the mesh-related complication rate after greater than 10 years of follow-up was greater than that noted during 5 years of follow-up, the complication rate was acceptable. No significant difference in outcomes were noted between repair surgery using the Prolift kit and self-cut Gynemesh.

## Data Availability

The datasets used and / or analyzed during the current study are available from the corresponding author on reasonable request.

## Abbreviations

POP: Pelvic organ prolapse
SUI: Stress urinary incontinence
TVM: Transvaginal mesh
PGI-I: The Patient Global Impression of Improvement
RCT: Randomized controlled trials
